# A novel antioxidant peptide from black soybean [*Glycine max* (L.) Merr.] with verified MPO interaction and multi-scale antioxidant activity

**DOI:** 10.3389/fnut.2025.1649684

**Published:** 2025-10-21

**Authors:** Dan Zhu, Hai Zheng, Xiaolin Xie, Bingqi Zhang

**Affiliations:** ^1^Department of Endocrinology and Clinical Nutrition, Gezhouba Central Hospital of Sinopharm, Third Clinical Medical College of China Three Gorges University, Yichang, China; ^2^Clinical College of Chinese Medicine, Hubei University of Chinese Medicine, Hubei Shizhen Laboratory, Hubei Provincial Hospital of Traditional Chinese Medicine, Wuhan, China; ^3^Xiangyang Central Hospital, Affiliated Hospital of Hubei University of Arts and Science, Xiangyang, China

**Keywords:** antioxidant peptides, black soybean, surface plasmon resonance, myeloperoxidase, molecular simulation

## Abstract

Oxidative stress-related diseases drive the demand for natural antioxidants, with plant-derived peptides emerging as promising candidates. This study focused on identifying and characterizing a novel antioxidant peptide from black soybean (Glycine max) aqueous extracts, targeting myeloperoxidase (MPO), a key enzyme in oxidative damage. Peptide components were profiled by liquid chromatography-tandem mass spectrometry (LC-MS/MS). The candidate peptide VPNHFNAP was selected via molecular docking and dynamics simulations against MPO, followed by binding affinity validation using surface plasmon resonance (SPR). Antioxidant capacity was assessed through DPPH/ABTS radical scavenging assays (IC50: 12.3 ± 0.8 μM and 9.7 ± 0.5 μM, respectively). Cytoprotective effects were evaluated in HaCaT cells under H_2_O_2_-induced oxidative stress (viability by MTT assay, ROS by DCFH-DA fluorescence). VPNHFNAP exhibited strong MPO binding (KD = 1.2 × 10^−7^ M, SPR) and radical scavenging activity (DPPH: 82.4 ± 3.1%; ABTS: 88.6 ± 2.7% at 100 μM). Molecular docking revealed hydrogen bonds with MPO’s His95 and Arg239. In cellular models, 50 μM VPNHFNAP increased viability by 2.1-fold (p < 0.001 vs. H_2_O_2_ group) and reduced ROS levels by 58 ± 4%. This study establishes VPNHFNAP as a potent MPO-targeted antioxidant peptide through an integrated computational-experimental strategy. Its dual function (direct radical scavenging + cellular protection) highlights potential applications in functional foods or cosmeceuticals. The screening framework also advances plant peptide discovery by combining bioinformatics with multi-level validation.

## 1 Introduction

Reactive oxygen species (ROS) are highly reactive molecules generated as byproducts of cellular metabolism, including hydrogen peroxide (H₂O₂), hydroxyl radicals (·OH), and others. Under physiological conditions, ROS play essential roles in various biological processes such as cell signaling, immune defense, and regulation of cell proliferation. However, when ROS production exceeds the capacity of the endogenous antioxidant defense system, oxidative stress occurs (1, 2). Excessive ROS can induce oxidative modifications of biomacromolecules, including lipid peroxidation, protein carbonylation, and DNA damage, ultimately disrupting cellular structure and function (3). Increasing evidence has demonstrated that oxidative stress is closely associated with the onset and progression of numerous chronic diseases. In particular, the excessive accumulation of ROS contributes to neuronal damage and promotes the development of neurodegenerative disorders such as Alzheimer’s disease, and also plays a key role in the pathogenesis of atherosclerosis by inducing endothelial dysfunction and inflammatory responses (4, 5).

Myeloperoxidase (MPO), an endogenous peroxidase enzyme, is predominantly expressed and secreted by activated neutrophils and plays a critical role in the host immune defense system (6). However, excessive activation of MPO can lead to the overproduction of ROS, overwhelming the body’s antioxidant defense mechanisms and contributing to the progression of various chronic diseases mentioned above. To counteract MPO-mediated oxidative damage, two major intervention strategies have been proposed: inhibition of MPO activity and scavenging of free radicals. MPO inhibitors can directly block its catalytic activity, thereby reducing the generation of harmful oxidative products, while their antioxidant potential can also contribute to redox homeostasis by directly scavenging already-formed ROS (7).

Black soybean [*Glycine max (L.) Merr.*], belonging to the family *Fabaceae*, subfamily *Faboideae*, genus *Glycine*, and subgenus *Soja*, is a traditional legume crop characterized by its black seed coat. It has been cultivated in China for thousands of years, and China remains the largest producer and consumer of soybeans globally, with substantial production and consumption of black soybean in particular (8). Black soybean exhibits a higher protein content and a more essential amino acid profile compared to yellow soybean, with protein levels ranging from 41.38 to 44.32%, making it a valuable resource for the development of bioactive peptides (9). Owing to its superior nutritional properties and associated health benefits—particularly its potent antioxidant activity—black soybean has garnered increasing attention. Several studies have reported that black soybean extracts possess significant antioxidant activities, including strong DPPH and ABTS radical scavenging abilities (10). In comparison with synthetic antioxidants such as butylated hydroxytoluene (BHT) and butylated hydroxyanisole (BHA), naturally derived antioxidant peptides are more readily absorbed and exhibit fewer adverse effects (11). Antioxidant peptides derived from black soybean have also been reported in previous studies. For example, Chen et al. performed ultrafiltration and RP-HPLC on black soybean processing by-products, and subsequently identified two peptides, LVPK and IVPK, which exhibited antioxidant activity and free radical scavenging capacity (12). Molecular docking is an in silico method used to predict the binding mode and affinity between a ligand and its target receptor, and to identify key interacting residues. The binding energy calculated from docking reflects the stability of the ligand–receptor complex, and a lower binding energy generally indicates stronger and more stable interactions, which can be used as a criterion for prioritizing candidate antioxidant peptides. Owing to its high efficiency and low cost, this technique is widely applied in screening bioactive peptides from natural products (13). For instance, Cai et al. identified two novel antioxidant peptides, ACGSDGK and KFCSGHA, from *Thunnus albacares* protein hydrolysates through molecular docking with MPO, demonstrating both strong binding affinity to the MPO protein and significant radical scavenging activity (14). Similarly, Gao et al. used LC–MS/MS to identify peptides from sturgeon ovarian protein hydrolysates and, based on molecular docking with MPO, screened out two novel antioxidant peptides, FDWDRL and FEGPPFKF, with demonstrated antioxidant bioactivity (15). However, despite the advantages of computational approaches, limitations remain when it comes to simulating physiological regulation and accurately modeling peptide–protein interactions. Many studies using molecular simulation to explore interactions between peptides and MPO proteins have primarily remained at the computational level, or have relied on indirect evidence—such as changes in cellular antioxidant enzyme levels (e.g., SOD, CAT)—to infer downstream biological effects potentially resulting from MPO inhibition by peptides, rather than providing direct experimental validation of peptide–MPO binding interactions (16–18).

In this study, black soybean was selected as the research subject due to its promising potential as a source of novel antioxidant peptides. A candidate peptide with the strongest predicted binding affinity to MPO was first screened through molecular docking, and its interaction was further evaluated using molecular dynamics simulation. The direct binding interaction between the synthetic peptide and MPO protein was then validated using SPR analysis. Finally, the antioxidant activity of the peptide was assessed through chemical antioxidant assays and cellular experiments, in order to further investigate the antioxidant potential of black soybean and elucidate the underlying mechanism of action of its antioxidant peptides.

## 2 Materials and methods

### 2.1 Materials

Black soybeans were purchased from the local market in Hubei Province, China. Ultrafiltration membranes were obtained from Shanghai Langji Co., Ltd. HaCaT cells were purchased from Dudi Biology (Shanghai, China). DPPH radical scavenging assay kits and ABTS radical scavenging capacity assay kits were purchased from Shanghai Yuanye Bio-Technology Co., Ltd. CCK-8 cell viability assay kits and ROS detection kits were obtained from Beyotime Biotechnology (China). The peptide used in this study was synthesized by C-Peptide (Wuhan) Biotechnology Co., Ltd., and its molecular weight and purity (≥98%) were confirmed by HPLC and UPLC–MS/MS analysis. Consumables used in cell experiments were purchased from Nest Biotechnology, Biosharp, and GIBCO.

### 2.2 Pretreatment of black soybeans

The pretreatment of black soybeans was performed following the method described by Huang et al., with some modifications (19). Black soybeans were thoroughly washed and soaked in water for 8 h. A total of 250 g of soaked beans were mixed with water at a solid-to-liquid ratio of 1:3 and subjected to high-pressure cooking at 70 kPa and 121 °C for 1 h. After cooking, the broth and softened beans were separated. The cooked beans were then homogenized using a high-speed blender for 5 min and returned to the separated broth for an additional high-pressure cooking cycle under the same conditions (70 kPa, 121 °C, 1 h). The resulting mixture was filtered through coarse gauze to remove solid residues, and the filtrate was centrifuged at 10,000 rpm for 10 min at 4 °C to collect the supernatant.

The obtained supernatant was subjected to sequential ultrafiltration to isolate low-molecular-weight components. To prevent membrane damage, ultrafiltration was first performed using a 10,000 Da cutoff membrane, followed by further filtration with a 3,000 Da cutoff membrane. The final filtrate, containing peptides with molecular weights ≤3,000 Da, was concentrated by rotary evaporation and lyophilized to obtain the test sample powder.

### 2.3 Peptide identification by LC–MS/MS

Peptide identification was performed using a Thermo Scientific Ultimate 3,000 UHPLC system coupled with a Q Exactive™ HF-X Hybrid Quadrupole-Orbitrap mass spectrometer. The sample powder was reconstituted in 0.1% formic acid aqueous solution, followed by centrifugation at 10,000 rpm for 10 min at 4 °C. The supernatant was subjected to desalting using a solid-phase extraction column prior to LC–MS/MS analysis. Peptide separation was carried out on a C18 reversed-phase column. The mobile phase consisted of solvent A (0.1% formic acid in water) and solvent B (0.1% formic acid in acetonitrile). The mass spectrometer operated in data-dependent acquisition mode, with both MS1 and MS2 spectra collected. Raw data were analyzed using PEAKS Studio software, with peptide sequences identified by searching against the UniProt database combined with *de novo* sequencing. The identified peptides were further analyzed using Excel, and MS/MS spectra of the target peptides were exported from PEAKS for further analysis.

### 2.4 Molecular simulation

#### 2.4.1 Molecular docking of peptides with the MPO receptor

Molecular docking was performed following the method described by He et al. (20). The selected protein receptor was myeloperoxidase (MPO, PDB ID: 3F9P). The original crystal structure was processed using PyMOL to remove water molecules and small ligands. The cleaned structure was then converted from .pdb to .pdbqt format using AutoDockTools. Peptides identified with molecular weights less than 1,500 Da were modeled and subjected to energy minimization using ChemDraw3D. The optimized structures were then imported into AutoDockTools for torsion bond detection and file format conversion. Molecular docking was conducted using AutoDock Vina, with the docking grid covering the entire MPO receptor. For each peptide, nine docking conformations were generated by default, and the one with the lowest binding energy was selected to construct the peptide-receptor complex for further analysis. The resulting complex was then analyzed using PyMOL and Discovery Studio to visualize and characterize the receptor–ligand interactions.

#### 2.4.2 Molecular docking analysis between VPNHFNAP and DPPH/ABTS free radicals

The 3D structures of DPPH and ABTS were obtained in .sdf format from the PubChem database,^1^ with their respective compound IDs being 15,911 and 5,360,881. The ligand structures were converted to .pdbqt format and torsional bonds were defined using AutoDockTools. The selected peptides were prepared as receptors following the procedure described in Section 2.4.1, including conversion to .pdbqt format. Molecular docking between each peptide and DPPH or ABTS was performed separately, and the docking results were further analyzed accordingly.

#### 2.4.3 Molecular dynamics simulation

Molecular dynamics simulation was conducted with reference to the method of Aamir Mehmood et al., with some modifications (21). The peptide–MPO complex showing the strongest binding affinity from molecular docking (see section 2.4.1) was selected for simulation using GROMACS2022. The complex was topologized using UCSF Chimera and SwissParam, then placed in a cubic simulation box with a minimum distance of 10 Å from any atom to the box boundary. The system was solvated with flexible SPC water molecules, and Na^+^ and Cl^−^ ions were added to neutralize the total charge. After energy minimization using the steepest descent algorithm for 50,000 steps, the system underwent NVT and NPT equilibration, followed by a 25 ns production run. Root mean square deviation (RMSD), number of hydrogen bonds (HB), and solvent-accessible surface area (SASA) were analyzed, and the Gibbs free energy landscape was calculated using the g_sham tool and xpm2txt.py script. Visualization of results was performed using Origin.

### 2.5 Surface plasmon resonance assay

The receptor protein human myeloperoxidase (MPO) was purchased from Biodragon company and verified by SDS-PAGE to have a molecular weight consistent with the theoretical value predicted from its amino acid sequence, with a purity of ≥90%. The synthetic peptide VPNHFNAP was obtained from C-Peptide (Wuhan) Biotechnology Co., Ltd., and its molecular weight and purity (≥98%) were confirmed by HPLC and UPLC-MS/MS. SPR analysis was performed using a CM5 sensor chip. The surface of the carboxymethylated dextran matrix was activated using EDC/NHS chemistry, followed by immobilization of the MPO protein; a reference channel without MPO was used as the control. The peptide VPNHFNAP was diluted in running buffer to prepare a series of concentrations and injected at a flow rate of 20 μL/min. The association and dissociation phases were recorded, and after each injection cycle, the chip surface was regenerated with 10 mM glycine-HCl (pH 2.0). All experiments were conducted at 22 °C. Data were analyzed using Biacore T200 Evaluation Software to calculate the kinetic binding parameters.

### 2.6 DPPH and ABTS radical scavenging assays

Prior to the assay, the microplate reader was preheated for 1 h. The peptide samples were dissolved in sterile water to prepare a concentration gradient of 0.1, 1, 5, and 10 mg/mL. For the DPPH assay, 50 μL of sterile water and 450 μL of DPPH solution (0.1 mM in anhydrous ethanol) were added to the blank tube. For the control tube, 50 μL of peptide solution and 450 μL of anhydrous ethanol were mixed. For the sample tubes, 50 μL of peptide solution and 450 μL of DPPH solution were combined. All mixtures were incubated in the dark at room temperature for 30 min. Absorbance was measured at 517 nm using a UV–visible spectrophotometer.

For the ABTS assay, ABTS radical solution was prepared by mixing Reagent A and Reagent B (from the assay kit) at a 1:1 volume ratio, followed by incubation in the dark for 12 h. The resulting ABTS solution was diluted 20-fold with anhydrous ethanol before use. The blank, control, and sample solutions were prepared in the same manner as in the DPPH assay, followed by 30 min of dark incubation. Absorbance was measured at 734 nm. The radical scavenging activity was calculated using the formula reported by Thanyaporn Aursuwanna et al. (22).

### 2.7 HaCaT cell experiments

#### 2.7.1 Recovery, culture, and subculture of HaCaT cells

HaCaT cell recovery, culture, and subculture were performed with reference to the method described by Yusha Zi et al., with some modifications (23). For recovery, cryopreserved HaCaT cells were retrieved from liquid nitrogen storage and quickly thawed in a 37 °C water bath with gentle shaking. Just before complete thawing, the tube was removed from the water bath, and the cell suspension was immediately transferred into a 15 mL centrifuge tube containing 9 mL of pre-warmed complete medium. The complete medium consisted of DMEM supplemented with 10% fetal bovine serum (FBS) and 1% penicillin–streptomycin (P/S). Cells were centrifuged at 800 rpm for 5 min at 4 °C. After discarding the supernatant, the cell pellet was resuspended in 5 mL of fresh complete medium and transferred to a culture flask equipped with a ventilated cap. Cells were incubated in a humidified incubator at 37 °C with 5% CO₂ and 95% humidity.

For subculturing, when cell confluence reached approximately 80%, the medium was discarded, and the cells were gently rinsed three times with 2 mL of PBS. Then, 1 mL of 0.25% trypsin solution was added, and the flask was incubated at 37 °C for about 1 min. Once the cells became round and partially detached (as observed under a microscope), 3 mL of complete medium was added to terminate the digestion. The cell suspension was gently pipetted to disperse the cells and transferred to a 15 mL centrifuge tube. After centrifugation at 800 rpm for 5 min at 4 °C, the supernatant was discarded. The pellet was resuspended in fresh medium and seeded into new culture flasks at a ratio of 1:3, followed by incubation under the same culture conditions.

#### 2.7.2 CCK8 cell viability assay

After digestion and resuspension, HaCaT cells were seeded into a 96-well plate at a concentration of 1 × 10^^4^ cells/ml, with 100 μL of complete culture medium added to each well. Three wells with no cells were treated with an equal volume of complete culture medium as the blank group. The cells were incubated for 24 h to allow attachment. For the experiment, the control and blank groups were replaced with an equal volume of fresh medium, while the treatment groups were treated with a concentration gradient of 100, 200, 500, 1,000, and 1,500 μg/mL H_2_O_2_ and peptide samples. The H_2_O_2_ group was incubated for 1 h, while the peptide sample group was incubated for 24 h. After incubation, the original medium was discarded, and 100 μL of CCK8 working solution (prepared from 90% DMEM + 10% CCK8 reagent) was added to each well. The blank group, which contained no cells, was used as the reference. The plate was incubated for an appropriate period, and the absorbance at 450 nm was measured using a microplate reader. Cell viability was calculated using the following formula:

$\begin{array}{l} \text{Cell viability}\;(\%)=100\%\times ({\mathrm{OD}}_{\text{sample}}-{\mathrm{OD}}_{\text{blank}})/\\ ({\mathrm{OD}}_{\text{control}}-{\mathrm{OD}}_{\text{blank}}). \end{array}$

Based on the cell viability results corresponding to the concentration gradient, the experimental concentration was set to 500 μg/mL for H_2_O_2_ and 1,000 μg/mL for the peptide. In the experimental groups, the cells were first incubated with peptides for 24 h (the control group did not receive the peptide), and then H_2_O_2_ was added for 1-h incubation. After incubation, the original medium was removed, and 100 μL of CCK8 working solution was added. The absorbance was measured and cell viability was calculated under the same conditions.

#### 2.7.3 Quantification of ROS

After digestion and resuspension, HaCaT cells were seeded into a 6-well plate at a concentration of 1 × 10^4 cells/ml, with 2 mL of culture medium added to each well. The cells were incubated for 24 h to allow attachment and adaptation. After 24 h, the cells were divided into treatment groups. The control and blank groups were replaced with an equal volume of normal culture medium, while the treatment group was incubated with a 1,000 μg/mL peptide solution for 24 h. After that, both the control and treatment groups were treated with 500 μg/mL H2O2 and incubated for an additional 1 h.

After incubation, the medium was discarded, and DCFH-DA probe was diluted in serum-free medium at a 1:1000 ratio. Then, 1.5 mL of the diluted solution was added to each well, and the cells were incubated at 37 °C in the dark for 20 min. The blank group was treated with an equal volume of serum-free medium. After incubation, the probe solution was discarded, and the cells were washed by gently pipetting with PBS to detach them completely. The cell suspension was collected into a 1.5 mL centrifuge tube and centrifuged at 800 rpm/min for 5 min at 4 °C. The supernatant was discarded, and the cells were resuspended in 1 mL PBS solution. The fluorescence intensity was measured using a fluorescence spectrophotometer with an excitation wavelength of 488 nm and an emission wavelength of 525 nm. The total protein concentration of each sample was determined using the BCA method. The fluorescence value was then normalized to the fluorescence intensity per milligram of protein, and the results were statistically analyzed.

### 2.8 Statistical analysis

All experiments were independently repeated three times, and the data are presented as the mean ± standard deviation (Mean ± SD). Statistical analysis was performed using GraphPad Prism 9.0 software. For comparisons between groups, one-way analysis of variance (ANOVA) followed by Tukey’s *post-hoc* multiple comparison test was used. The significance levels were set as follows: *p* < 0.05 (*), *p* < 0.01 (**), *p* < 0.001 (***), *p* < 0.0001 (****).

## 3 Results and discussion

### 3.1 *in silico* screening of peptides via molecular simulation

#### 3.1.1 Molecular docking of identified peptides

A total of 3,121 peptides were identified from the <3,000 Da lyophilized fraction of the aqueous extract of black soybean using LC–MS/MS. To efficiently identify peptides with potential antioxidant activity, further screening was performed. According to previous reports, peptides with molecular weights below 1,500 Da generally exhibit stronger antioxidant activity than those above 1,500 Da, and most known antioxidant peptides consist of 2–10 amino acid residues (24, 25). Based on these criteria, 1,145 peptides from the initial 3,121 identified peptides met the requirements of both molecular weight and sequence length (Supplementary Table 1).

**Figure 1:**
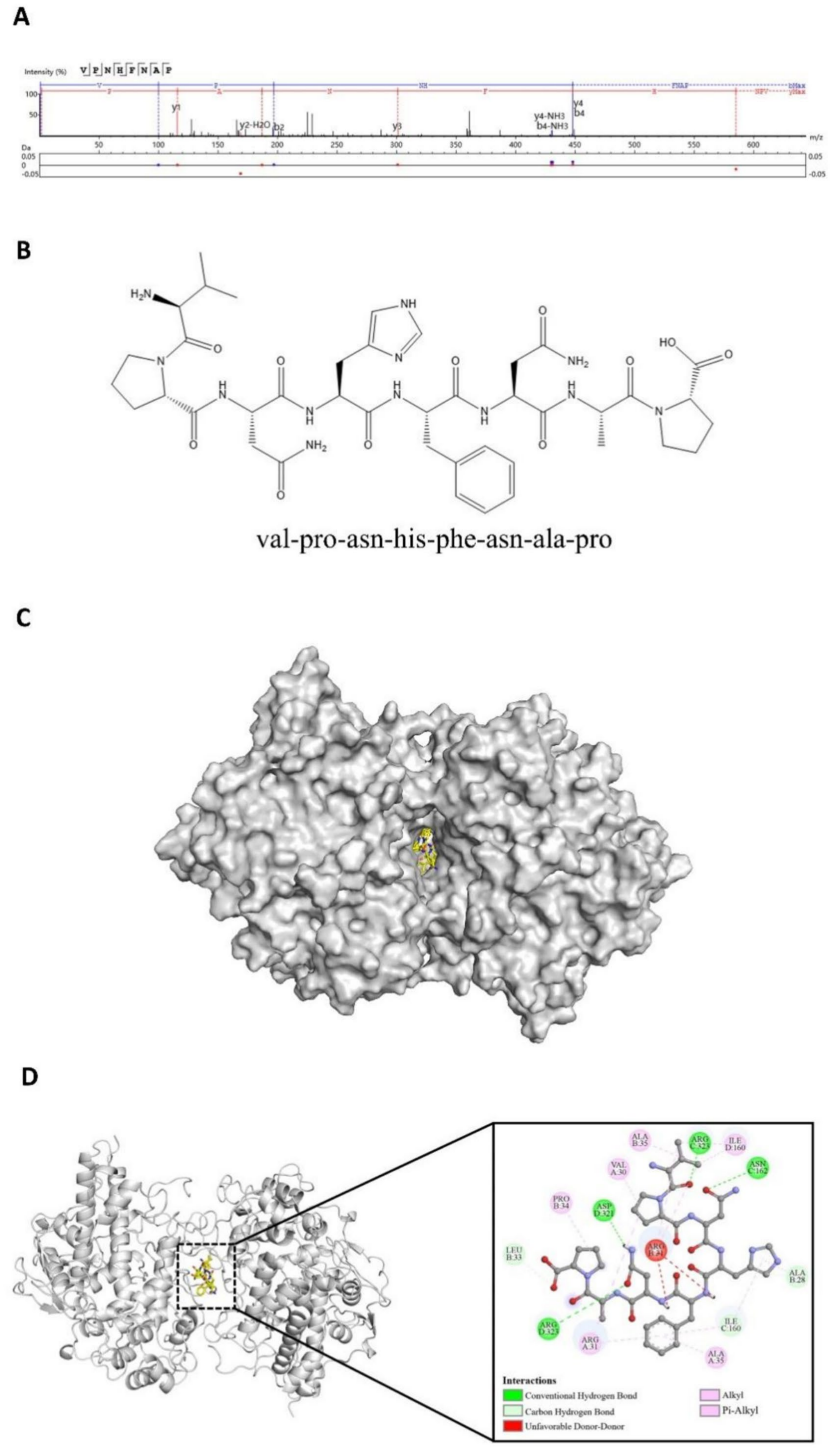
Identification and molecular docking of the target peptide VPNHFNAP. **(A)** MS/MS spectrum of VPNHFNAP. **(B)** 2D chemical structure of VPNHFNAP. **(C)** Overview of the docking complex between VPNHFNAP and MPO receptor protein. **(D)** Detailed interaction between VPNHFNAP and MPO, showing the MPO receptor as a gray cartoon structure and VPNHFNAP as yellow sticks.

Peptides can inhibit the catalytic activity of myeloperoxidase (MPO) by occupying its active site or blocking its access channel, thereby reducing the generation of strong oxidants such as hypochlorous acid (HOCl). This inhibition leads to a decrease in MPO-dependent reactive oxygen species (ROS) bursts, thereby alleviating oxidative stress damage caused by excessive ROS, and such peptides often exhibit significant antioxidant activity (26, 27). A total of 1,145 peptides were subjected to molecular docking modeling and individually docked with the MPO receptor protein. Peptides with lower binding energy are generally considered to have a stronger interaction with the target protein and are more likely to trigger downstream biological effects (28). Based on the docking scores, the peptides were ranked according to their binding energy with MPO. The peptide VPNHFNAP exhibited the strongest binding affinity, with a docking energy of −12.04 kcal/mol. This peptide was therefore selected as a potential potent antioxidant peptide that may act as an MPO inhibitor. Its MS/MS fragmentation spectrum and 2D chemical structure are shown in Figures 1A,B, respectively.

A detailed analysis of the molecular docking results revealed that VPNHFNAP successfully docked into the core pocket of the MPO receptor protein (Figure 1C), occupying the same active site as antioxidant peptides previously identified from hemp seed protein (29). VPNHFNAP formed multiple interactions with surrounding amino acid residues at the binding site, involving five types of intermolecular forces: conventional hydrogen bonds, carbon hydrogen bonds, alkyl interactions, pi-alkyl interactions, and unfavorable donor–donor interactions.

Specifically, conventional hydrogen bonds were formed with ASN162 and ARG323 of chain C, and ASP321 and ARG323 of chain D. Carbon hydrogen bonds were observed with ALA28 and LEU33 of chain B, and ILE160 of chain C. Alkyl and pi-alkyl interactions (categorized as hydrophobic interactions) were formed with VAL30, ARG31, and ALA35 of chain A, PRO34 and ALA35 of chain B, and ILE160 of chain D. Additionally, an unfavorable donor–donor interaction was observed between VPNHFNAP and ARG31 of chain B (Figure 1D). This type of unfavorable interaction may slightly weaken the overall binding efficiency. As reported by Sidali Zaidi et al., a similar unfavorable donor–donor interaction was observed between myricetin-galactoside and an ARG residue in MPO. The guanidinium side chain of ARG contains multiple hydrogen bond donors, and when in proximity to other strong donor groups such as –OH or –NH₂, repulsive electronic interactions may occur, leading to such unfavorable effects. Nevertheless, their reported docking energy was still as low as −8.9 kcal/mol, suggesting that favorable interactions elsewhere in the binding site can compensate for the unfavorable forces (30).

Through the synergistic effect of multiple intermolecular interactions, VPNHFNAP formed a stable binding conformation within the catalytic pocket of the MPO receptor. Notably, the interaction mode observed here is highly consistent with the binding patterns of antioxidant peptides derived from Atlantic sea cucumber, where conventional hydrogen bonding and hydrophobic interactions are identified as the primary driving forces contributing to the peptide–MPO binding (31). It is worth highlighting that this binding pattern is not exclusive to peptides from sea cucumber. Similar interaction modes have also been reported for antioxidant peptides derived from glycinin and conglycinin (32). In particular, the carbonyl oxygen (C=O) on the peptide backbone and –NH₂ side chains of the amino acids tend to form strong hydrogen bonds with the MPO receptor. These hydrogen bonds not only facilitate specific molecular recognition between the peptide and the MPO protein, but also contribute to the thermodynamic stability of the peptide–receptor complex (33). Furthermore, recent studies on antioxidant peptides identified from foxtail millet prolamin have demonstrated that hydrogen bonding interactions are the major contributors to their effective binding with MPO (32).

#### 3.1.2 Molecular dynamics simulation of the VPNHFNAP-MPO complex

To validate the molecular docking results and assess the potential induced-fit effects on the stability of the VPNHFNAP-MPO complex, a 25 ns molecular dynamics simulation was performed. This approach aimed to overcome the limitations of semi-flexible docking, in which the receptor is typically treated as rigid, thereby enabling a more comprehensive evaluation of the dynamic stability of protein–peptide interactions (18).

**Figure 2:**
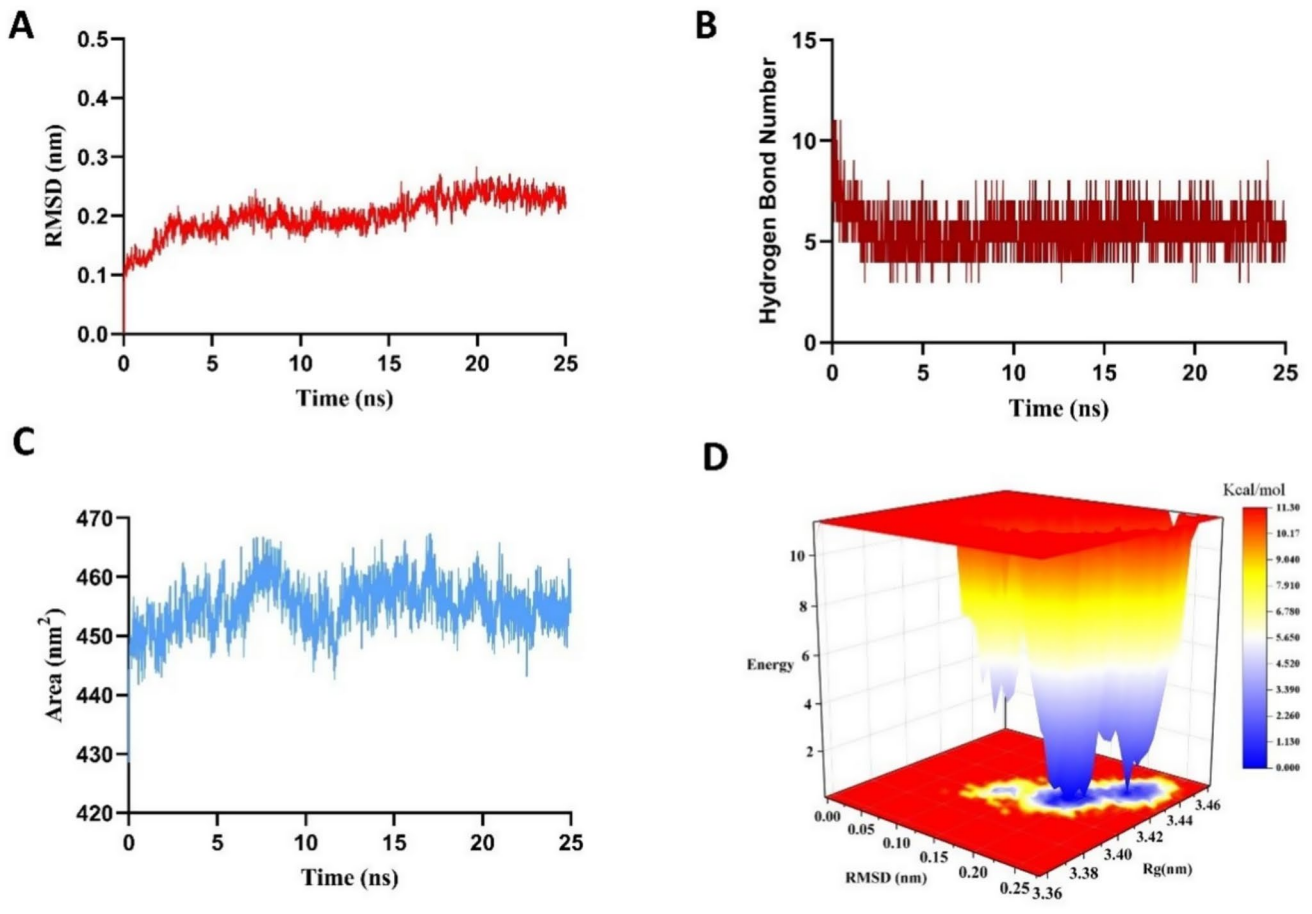
Results of a 25 ns molecular dynamics simulation of the VPNHFNAP–MPO complex. **(A)** Root-mean-square deviation (RMSD). **(B)** Number of hydrogen bonds. **(C)** Solvent-accessible surface area (SASA). **(D)** Three-dimensional Gibbs free energy landscape.

The root mean square deviation (RMSD) value reflects the deviation of the complex from its initial conformation over the course of the simulation. RMSD fluctuations are directly indicative of conformational stability, with deviations below 0.5 nm generally considered acceptable (34). As shown in Figure 2A, the RMSD displayed significant fluctuations during the initial phase (0–5 ns), likely due to induced-fit adjustments between MPO and VPNHFNAP. However, after 5 ns, the RMSD stabilized around 0.23 ± 0.057 nm, suggesting the complex had reached a dynamically equilibrated and structurally stable state. Hydrogen bonds, as key driving forces in protein-peptide interactions, offer insights into binding stability (35). The number of hydrogen bonds formed during the simulation closely paralleled the RMSD trend (Figure 2B), initially decreasing during 0–5 ns, followed by stabilization at approximately 5 ± 1 hydrogen bonds. This indicates the formation of a stable interaction network between VPNHFNAP and the MPO receptor during the equilibrium phase. A comparable simulation conducted on the antioxidant peptide LYSPH, derived from cherry seeds, revealed a docking binding energy of −7.9 kcal/mol, 3 ± 1 hydrogen bonds, and an RMSD fluctuation of ±0.047 nm (36), showing similar patterns to those found in this study.

The solvent-accessible surface area (SASA) further illustrated the dynamic binding behavior and solvent exposure of the complex. As shown in Figure 2C, SASA value increased significantly during the early simulation stage, likely due to two main factors: (1) the bulky hydrophobic aromatic ring of VPNHFNAP may not have fully penetrated the hydrophobic core of MPO owing to steric hindrance and hydrophobic effects, leaving portions exposed to solvent (37); and (2) the formation of an initial hydration layer due to strong interactions between polar residues on the MPO surface and surrounding water molecules (38). As the simulation progressed, both protein and peptide conformations adjusted, and solvent distribution around the protein became more uniform, ultimately leading the system to equilibrium. Although the final SASA remained approximately 5 nm^2^ higher than the initial value, its low fluctuation range confirmed the relative stability of the complex.

To further evaluate the thermodynamic profile of the complex, the g_sham module of GROMACS2022 was used to calculate the Gibbs free energy landscape based on RMSD and radius of gyration (Rg) values, generating a 3D free energy landscape (Figure 2D). In this surface, blue to white regions indicate low free energy value, whereas yellow to red regions reflect higher energy states. A scattered, rugged energy surface suggests weak or unstable interactions, whereas a single, smooth, concentrated energy basin represents strong and stable interactions (39). For the MPO–VPNHFNAP complex, the energy projection was focused in a small, defined region on the lower right of the plot, with a distinct and well-defined energy basin, indicating a thermodynamically stable binding conformation. This observation was consistent with the RMSD, hydrogen bond, and SASA results. Similarly, in a study conducted by Yan et al., antioxidant peptides GWY and QWY exhibited RMSD fluctuations of approximately 0.19 ± 0.01 nm during 25 ns simulations, and their corresponding free energy landscapes displayed well-confined energy basins within strict boundaries (40). Taken together, these results indicate that VPNHFNAP forms a stable intermolecular interaction network within the MPO binding pocket, resulting in a thermodynamically favorable binding conformation characterized by low Gibbs free energy. These findings highlight the potential of VPNHFNAP as a strong MPO-binding antioxidant peptide.

### 3.2 SPR analysis of the interaction between VPNHFNAP and MPO protein

**Figure 3:**
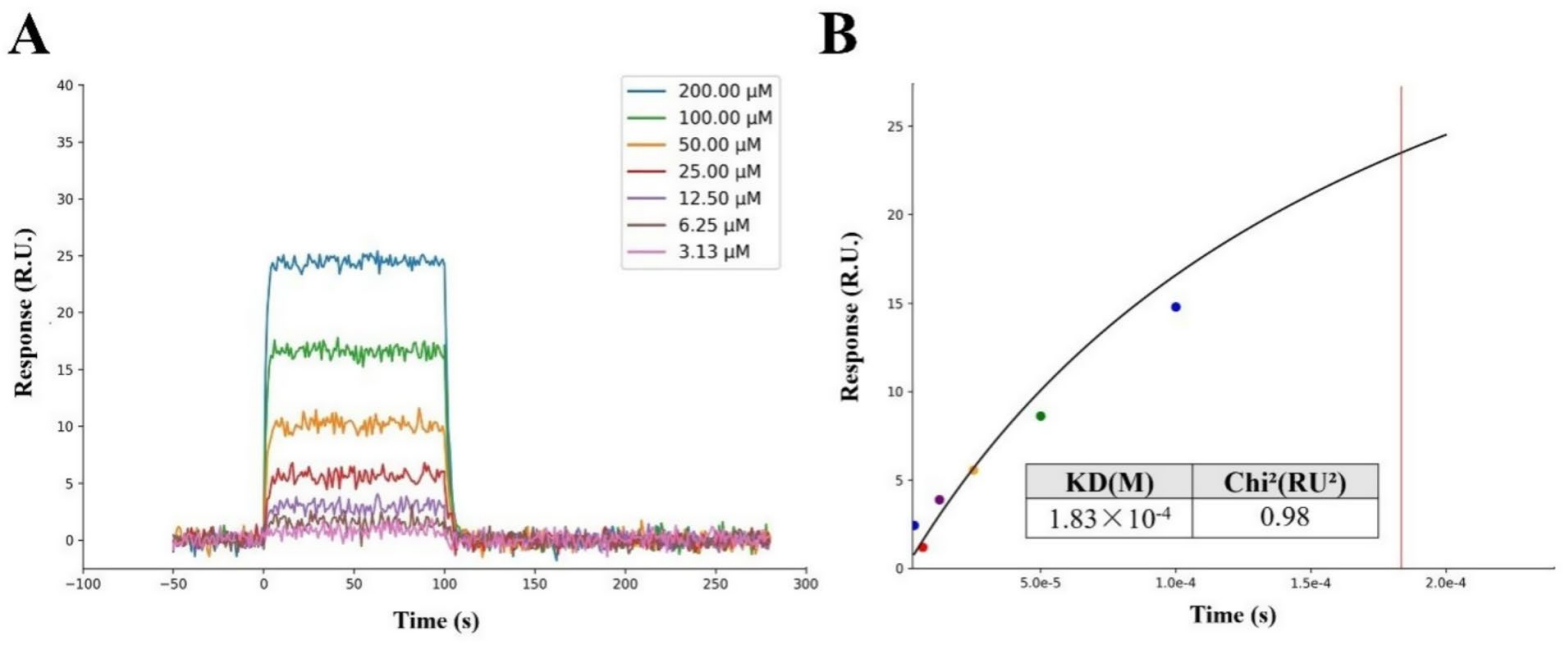
SPR analysis of the interaction between the peptide VPNHFNAP and the MPO receptor protein. **(A)** The response signals of VPNHFNAP at different concentrations. **(B)** Steady-state fitting diagram of VPNHFNAP binding to MPO. The KD and Chi^2^ values were calculated based on the fitted response curve.

To further validate the molecular simulation results and clarify the biological activity of VPNHFNAP, a SPR experiment was conducted using purified human MPO receptor protein and synthetic peptide VPNHFNAP. As shown in Figure 3A, the response unit (RU) values increased progressively with peptide concentrations ranging from 3.13 μM to 200 μM, indicating a dose-dependent binding affinity between VPNHFNAP and MPO. The RU values increased from approximately 0.79 RU to around 24.51 RU with increasing concentrations. The binding and dissociation processes were both rapid, suggesting that the molecular recognition and association between VPNHFNAP and MPO are facilitated by favorable intermolecular interactions, as previously observed in molecular simulations. Similarly, Bakytzhan et al. investigated the interaction between MPO and ceruloplasmin (Cp), a physiological MPO inhibitor, using SPR, and further explored Cp-derived peptides for their MPO-binding potential. Their results also demonstrated clear dose-dependent binding effect (41).

The kinetic fitting parameter Chi^2^ (RU^2^) was used to evaluate the quality of the fitting, with lower values indicating better agreement between the fitted curve and experimental data. Typically, a Chi^2^ value below 10RU^2^ is considered indicative of a reliable fit (42). In this study, the Chi^2^ value for the interaction between VPNHFNAP and MPO was 0.98RU^2^, suggesting a good fitting quality. The dissociation constant (KD) was determined to be 1.83 × 10^−4^ M (Figure 3B), indicating a moderate binding affinity. Notably, De Jong et al. employed SPR to investigate the binding interaction between staphylococcal peroxidase inhibitory protein (SPIN) and MPO. Compared to VPNHFNAP, SPIN is a larger molecule with a rigid *α*-helical framework and an N-terminal *β*-hairpin structure that deeply inserts into the MPO active site, forming a “molecular plug” that sterically hinders substrate molecules such as H₂O₂ from accessing the MPO catalytic site. The KD value for the SPIN–MPO interaction was reported as 1 × 10^−8^ M, which reflects a typical high-affinity antigen–antibody interaction (43). Although the KD value of VPNHFNAP is higher than that of SPIN, indicating a lower affinity, it still falls within the acceptable range for small-molecule drug candidates. This is consistent with the molecular simulation results, which predicted a favorable interaction between VPNHFNAP and MPO, supporting the hypothesis that VPNHFNAP may exert its inhibitory effect by binding to the active site of MPO and interfering with its catalytic activity (20).

Gao et al. identified key antioxidant peptides from hemp seed that exhibited free radical scavenging and cytoprotective effects against oxidative stress. These peptides were shown to bind tightly to the active site pocket of MPO, suggesting that VPNHFNAP may exert similar antioxidant effects through a comparable binding mechanism (29). The antioxidant activity of peptides is strongly influenced by their length, amino acid composition, and sequence (44). It has been reported that antioxidant peptides often contain hydrophobic residues such as G, A, V, L, I, P, F, and M (45), Notably, five out of the eight residues in VPNHFNAP are hydrophobic, accounting for 62.5% of the sequence. Additionally, Wen et al. demonstrated that the presence of V(Val) at either the N- or C-terminus significantly enhances antioxidant activity (46), while the inclusion of aromatic amino acids like F(Phe) has also been associated with improved radical scavenging potential (47). Taken together, these features also suggest that VPNHFNAP possesses strong potential as an effective antioxidant peptide.

### 3.3 Free radical scavenging assay

To directly characterize and verify the antioxidant activity of VPNHFNAP, *in vitro* free radical scavenging assays were performed based on the method described by Luan et al., with some modifications (48). Glutathione (GSH), a widely recognized antioxidant peptide, was employed as a positive control to evaluate the antioxidant potential of VPNHFNAP (49).

#### 3.3.1 DPPH and ABTS radical scavenging activities

**Figure 4:**
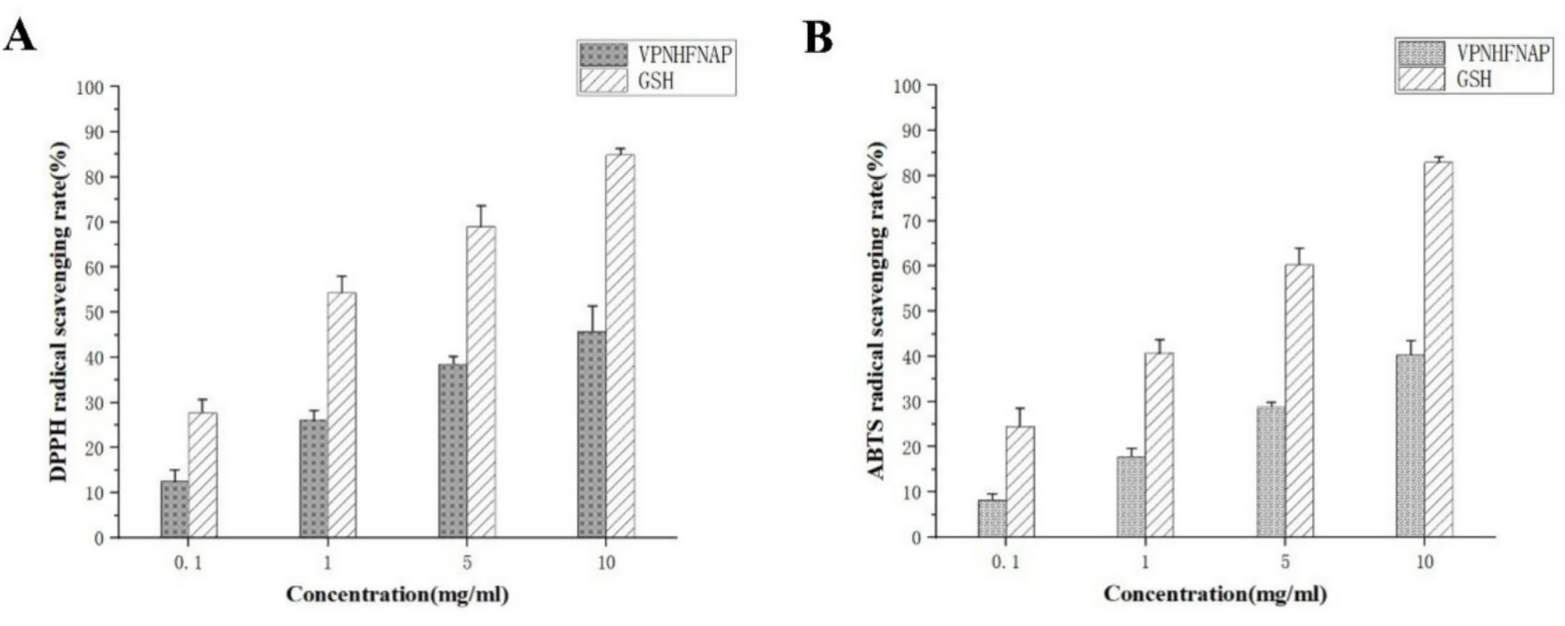
Free radical scavenging activity of VPNHFNAP, with GSH as the positive control. **(A)** DPPH radical scavenging activity. **(B)** ABTS radical scavenging activity.

As shown in Figure 4A, in the DPPH radical scavenging assay, VPNHFNAP exhibited dose-dependent scavenging activity, with scavenging rates of 12.48 ± 2.62%, 26.23 ± 1.97%, 38.59 ± 1.65%, and 45.85 ± 5.55% at concentrations of 0.1, 1, 5, and 10 mg/mL, respectively. In comparison, GSH displayed scavenging rates of 27.72 ± 2.86%, 54.36 ± 3.61%, 69.01 ± 4.68%, and 84.87 ± 1.39% at the corresponding concentrations. Similarly, as illustrated in Figure 4B, VPNHFNAP showed increasing ABTS radical scavenging activity with rising concentrations, with scavenging rates of 8.3 ± 1.28%, 17.79 ± 1.86%, 28.82 ± 1.03%, and 40.4 ± 3.19% at 0.1, 1, 5, and 10 mg/mL, respectively., GSH achieved scavenging rates of 24.52 ± 4.01%, 40.76 ± 3.01%, 60.28 ± 3.58%, and 82.92 ± 1.27% at the same concentrations. Both VPNHFNAP and GSH demonstrated a clear dose–response trend in their radical scavenging capacities. Notably, VPNHFNAP consistently achieved approximately half the scavenging efficiency of GSH at equivalent concentrations, suggesting that VPNHFNAP possesses appreciable free radical scavenging activity.

Similarly, Zhang et al. identified two antioxidant peptides, VVFVDRL and VIYVVDLR, from *Glycine max* hydrolysate, both of which contain N-terminal valine residues. At a concentration of 1 mg/mL, their DPPH and ABTS radical scavenging rates reached approximately 40 and 50%, respectively, which are markedly higher than those of VPNHFNAP (26.23 ± 1.97% and 17.79 ± 1.86%) at the same concentration (25). This enhanced activity was attributed to the presence of repeating amino acids within the peptide sequences, a feature that has also been associated with stronger bioactivity in antioxidant peptides derived from *Parkia speciosa* seeds (50). In addition, Fu et al. reported an antioxidant peptide, VLIPMP, from foxtail millet prolamin, which also features valine at the N-terminus and proline at the C-terminus. This peptide exhibited strong binding affinity to MPO in molecular docking studies, mainly via hydrogen bonding and other intermolecular interactions. Molecular dynamics simulations further confirmed the stability of the VLIPMP–MPO complex. In radical scavenging assays, VLIPMP showed notable activity, with IC₅₀ values of 4.45 mg/mL (DPPH) and 2.33 mg/mL (ABTS). Moreover, its binding free energy calculated by MM-PBSA was −40.83 ± 7.64 kcal/mol, and the number of hydrogen bonds formed with MPO was higher than that observed for VPNHFNAP (51).

Despite being derived from similar plant seed sources and possessing structurally related features, antioxidant peptides such as VLIPMP and VPNHFNAP exhibit significant differences in their MPO-binding affinities and radical scavenging capacities. These disparities may be attributed to differences in amino acid composition, highlighting the importance of sequence-specific features in determining antioxidant efficacy and warranting further investigation.

#### 3.3.2 Molecular docking of VPNHFNAP with free radicals

**Figure 5:**
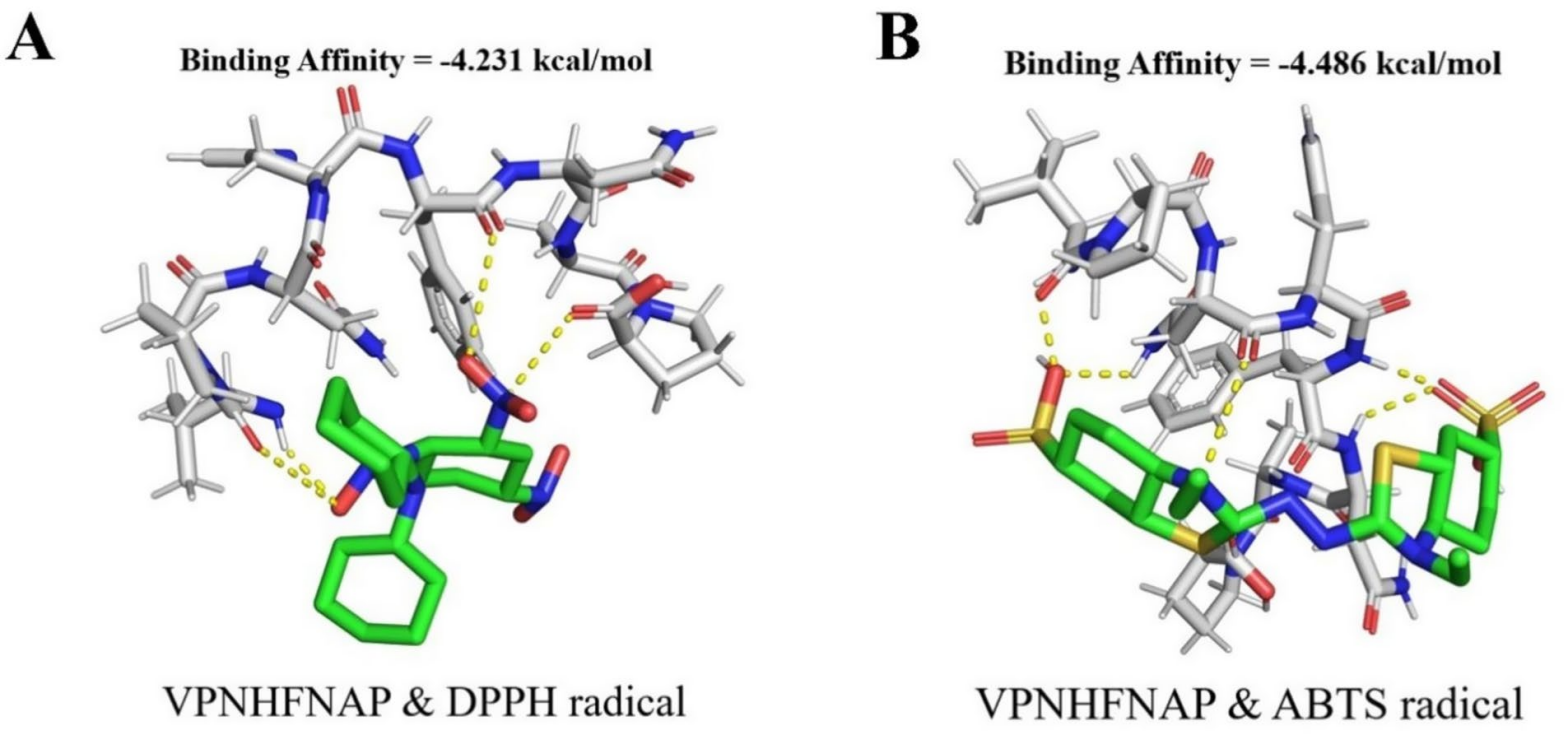
Molecular docking results of VPNHFNAP with free radical molecules. Green sticks represent free radical molecules, gray sticks represent VPNHFNAP, and yellow dashed lines indicate hydrogen bond interactions. **(A)** VPNHFNAP with DPPH radical. **(B)** VPNHFNAP with ABTS radical.

Following the observation of the free radical scavenging activity of VPNHFNAP, molecular docking studies were conducted to elucidate the potential mechanisms underlying its interactions with DPPH and ABTS radicals. As shown in Figure 5, VPNHFNAP formed strong interactions with both radical species. The docking binding energies with DPPH and ABTS were −4.231 kcal/mol and −4.486 kcal/mol, respectively. Similarly, Wang et al. employed AutoDock Vina to investigate the interactions between antioxidant peptides and free radicals, and reported that the binding energies of 16 antioxidant peptides with DPPH ranged from −3.3 to −4.4 kcal/mol, and with ABTS ranged from −3.2 to −4.7 kcal/mol (52). The binding energies of VPNHFNAP with DPPH and ABTS in the present study fall within this reported range, indicating comparable interaction strength. The strong binding affinities observed in the docking simulations are consistent with the dose-dependent radical scavenging activity of VPNHFNAP demonstrated in experimental assays. These results support the hypothesis that VPNHFNAP exerts its antioxidant effects by forming stable complexes with DPPH and ABTS radicals, thereby scavenging them.

### 3.4 Cellular antioxidant activity assay using H₂O₂-induced HaCaT cells

#### 3.4.1 Concentration screening for H₂O₂ and VPNHFNAP in HaCaT cells

Following the demonstration of free radical scavenging activity in chemical assays, the antioxidant activity of VPNHFNAP was further evaluated using a cell-based model. An oxidative stress model was established by H₂O₂-induced damage in HaCaT cells. As immortalized human keratinocytes, HaCaT cells possess a complete oxidative stress signaling pathway and a stable expression profile of antioxidant enzymes, making them an ideal model widely used in studies assessing the protective effects of antioxidant peptides (53, 54). Excessive concentrations of synthetic peptides may cause cytotoxicity (55), and the degree of cellular damage induced by H₂O₂ also varies with its concentration. To determine appropriate experimental concentrations for H₂O₂ and VPNHFNAP, we first investigated the effects of their respective concentrations on cell viability.

**Figure 6:**
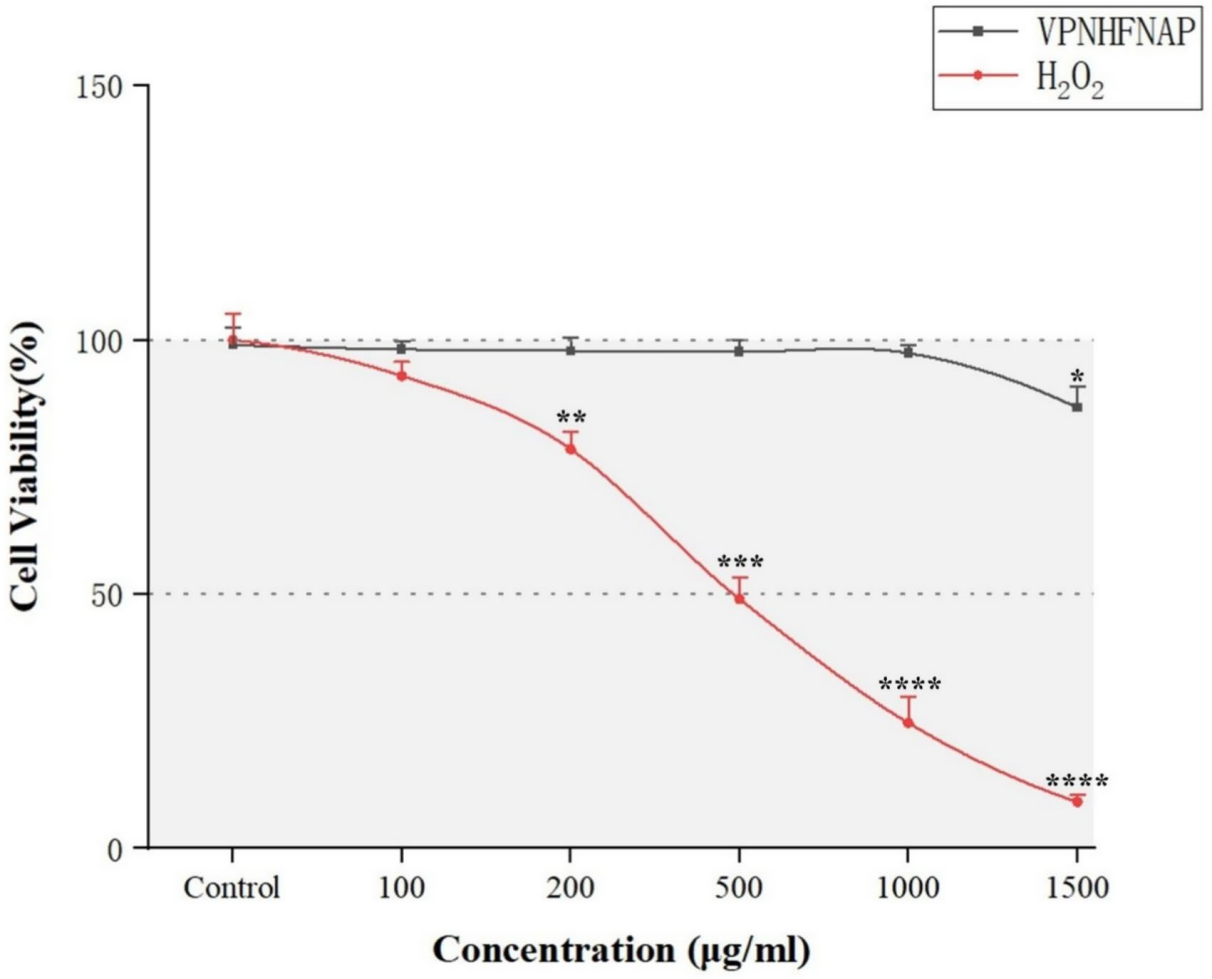
Line chart showing the effects of different concentrations of VPNHFNAP and H₂O₂ on the viability of HaCaT cells.

As shown in Figure 6, with increasing concentrations of H₂O₂, cell viability gradually decreased compared to the control group. A significant reduction (*p* < 0.01) was observed at 200 μg/mL, and over 90% of the cells were dead at 1500 μg/mL. At 500 μg/mL, the cell viability was 49.05 ± 4.24%, which was considered suitable for subsequent peptide activity evaluation (56). Therefore, 500 μg/mL H₂O₂ was selected as the modeling concentration for inducing oxidative damage. For VPNHFNAP, concentrations ranging from 100 to 1,000 μg/mL did not cause significant changes in cell viability compared to the control group, whereas a marked decrease was observed at 1500 μg/mL. Thus, 1,000 μg/mL was chosen as the treatment concentration for evaluating antioxidant effects. Similarly, Huang et al. reported that antioxidant peptides exhibited no significant toxic effects on HepG2 cells within the 25–1,000 μg/mL concentration range (57).

#### 3.4.2 Effects of VPNHFNAP on cell viability and ROS levels

**Figure 7:**
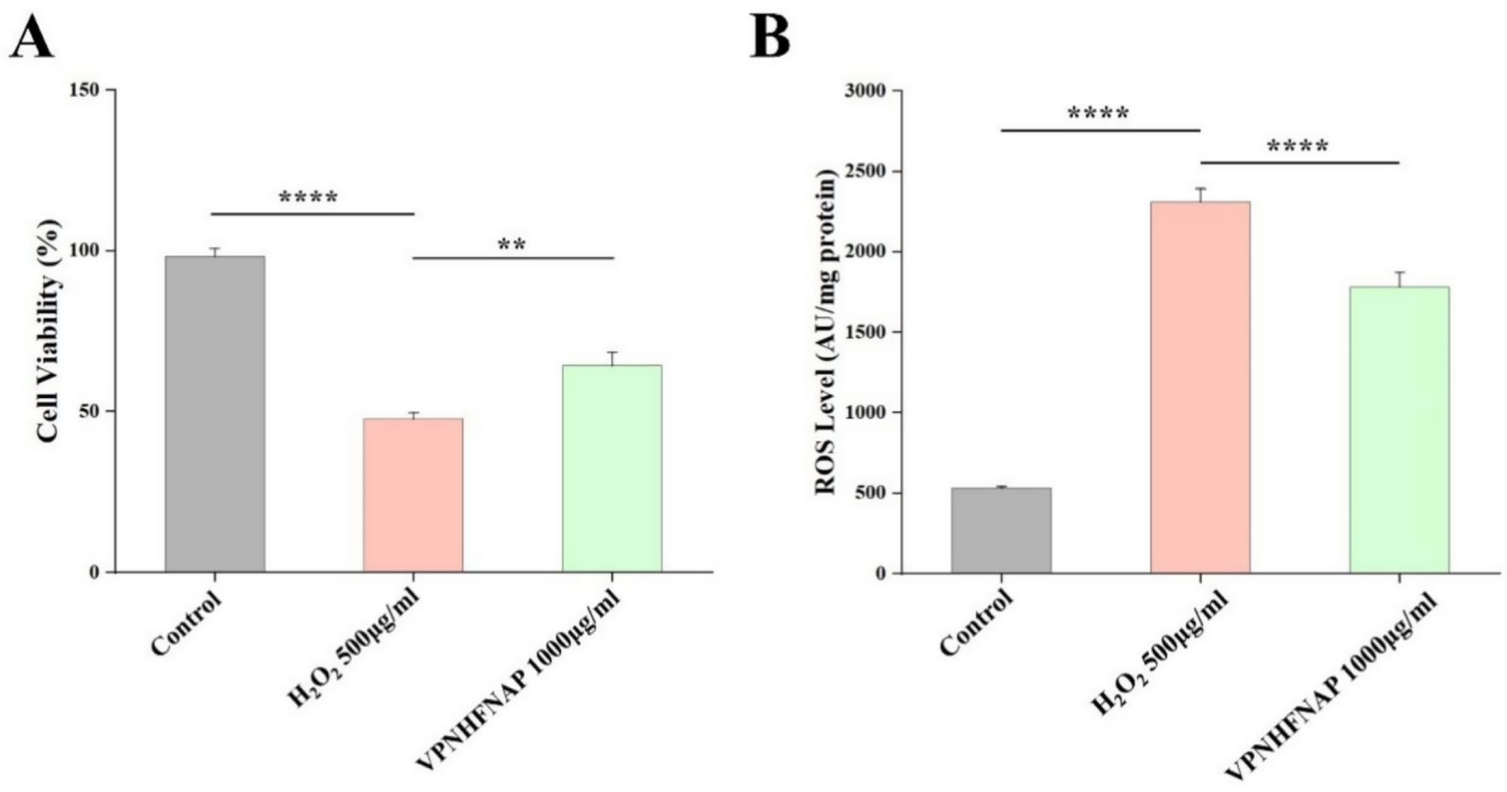
Effect of VPNHFNAP on H2O2-induced oxidative damage in HaCaT cells. **(A)** Cell viability of HaCaT cells. **(B)** ROS levels in HaCaT cells.

To investigate the protective effect of VPNHFNAP against oxidative damage, we evaluated the influence of VPNHFNAP on cell viability in HaCaT cells subjected to H₂O₂-induced oxidative stress. As shown in Figure 7A, treatment with 500 μg/mL H₂O₂ significantly decreased cell viability to 47.82 ± 1.87% compared with the control group (*p* < 0.0001). However, cells pretreated with 1,000 μg/mL VPNHFNAP exhibited a markedly higher viability of 64.38 ± 4.22%, indicating a significant improvement compared to the H₂O₂ group (*p* < 0.01). Oxidative stress leads to intracellular accumulation of ROS, and the level of ROS is a key indicator of oxidative damage. As shown in Figure 7B, the ROS level increased significantly from 541.25 ± 25.03 AU in the control group to 2128.33 ± 127.37 AU after treatment with 500 μg/mL H₂O₂ (*p* < 0.0001). In contrast, cells treated with VPNHFNAP showed a significantly reduced ROS level of 1863.16 ± 193.37 AU compared to the H₂O₂ group (*p* < 0.0001).

These results indicate that H₂O₂ treatment significantly elevated ROS levels in HaCaT cells, disrupting redox homeostasis and leading to pronounced oxidative stress, as evidenced by decreased cell viability (*p* < 0.0001). Conversely, VPNHFNAP effectively attenuated H₂O₂-induced oxidative damage, significantly improving cell viability (*p* < 0.01). The protective effect of VPNHFNAP may be attributed to its amino acid composition. The presence of hydrophobic residues such as V, P, F facilitates interaction with the hydrophobic regions of the cell membrane lipid bilayer, potentially enhancing cellular uptake of the peptide (58). Additionally, these amino acids may contribute to antioxidant activity through proton donation, thereby terminating free radical chain reactions. Aromatic amino acids, particularly Pro, are also considered antioxidant residues, capable of directly donating electrons to electron-deficient radicals, further enhancing the peptide’s antioxidant potential (59).

## 4 Conclusion

In this study, peptide components from aqueous extracts of black soybean were identified using LC–MS/MS. A novel antioxidant peptide, VPNHFNAP, was screened by targeting myeloperoxidase (MPO) protein through molecular docking combined with molecular dynamics simulations. Surface plasmon resonance (SPR) analysis confirmed the specific binding affinity of VPNHFNAP to MPO, validating the computational prediction results. *In vitro* chemical assays demonstrated that VPNHFNAP possesses significant DPPH and ABTS radical scavenging activity, and its underlying mechanism was further elucidated through molecular docking studies. In a HaCaT cell model of oxidative stress induced by H₂O₂, VPNHFNAP showed potent cytoprotective effects, significantly improving cell viability and reducing intracellular ROS levels. This work provides a theoretical basis for the high-value utilization of black soybean by-products, expands the current database of plant-derived antioxidant peptides, and proposes a screening strategy for antioxidant peptides based on the integration of computational simulation, molecular interaction validation, and biological evaluation.

## Data Availability

The original contributions presented in the study are included in the article/Supplementary material, further inquiries can be directed to the corresponding author.

## Footnotes

^1^ https://pubchem.ncbi.nlm.nih.gov/
